# The New York Institute of Stomatology

**Published:** 1897-01

**Authors:** 

**Affiliations:** The New York Institute of Stomatology


### THE NEW YORK INSTITUTE OF STOMATOLOGY.

A REGULAR meeting of the Institute was held on Tuesday evening, October 6, 1896, at the house of Dr. C. A. Woodward, 49 West Forty-sixth Street, the President, Dr. Benjamin Lord, in the chair.

*The President.*—This being the first meeting after our summer vacation, it is naturally an occasion for congratulations and greetings, but we have also feelings of sorrow and sadness, owing to the fact that death has visited us and taken from among us our secretary, Dr. C. F. Ives.

We can only think of him as a warm-hearted friend, an interesting associate, and a faithful and efficient officer, one who was greatly interested in the work of the Institute, and we shall greatly miss both his companionship and his valuable services.

It will be most fitting and proper that a committee be chosen to prepare and present, at a proper time, a suitable memorial in behalf of the deceased, to be entered upon our minutes and to go into our proceedings. What is the pleasure of the meeting in regard to it?

*Dr. George S. Allan.*—Mr. President, we are, of course, of one mind and one thought upon this matter. Dr. Ives was a man deeply interested in our work, and we shall miss him the more as we come to know how much he was worth to us; especially those of us who knew how patiently he bore up under his physical suffering and who knew the pathetic story of his agony at times. He breathed no word of fault-finding or railing against the burdens that became at last too heavy. Whoever met him, whether in the office, in the street, or in society, always found him the same pleasant, genial associate. It will not do to take up our time this evening to pass upon his merits in words of eulogy, but your suggestion, Mr. President, is a proper and fitting one; and I would move that a committee be appointed to put in proper shape our thoughts and feelings, and to report at an early meeting.

Dr. Allan's motion was carried, and the President appointed, as such committee, Dr. Howe and Dr. Davenport.

The President requested Dr. Wilson to act as secretary for the evening.

The minutes of the last meeting were read and approved.

*The President.*—Dr. Davenport, has a communication to offer and we will be glad to hear from him now.

*Dr. S. E. Davenport.*—Intelligence of the death of Dr. E. A. Stebbins, of Shelburne Falls, Massachusetts, having recently been received, I beg the privilege of paying a short tribute to his sterling qualities and fine professional attainments.

A successful practitioner and an exceptionally fine operator, his fame was established throughout the profession because of original work done in two important directions,—carefully tabulated results of experiments concerning the relative value, strength, and durability of different materials and methods used in prosthetic dentistry, and the establishment of the use of nitrate of silver in daily practice on a scientific basis.

Nitrate of silver has, of course, long been used for cauterizing sensitive and eroded surfaces, but to Dr. Stebbins more than any other man does the profession owe its present knowledge of the great value of the nitrate of silver as an agent for the prevention and limitation of caries.

He made extensive experiments with it for a period of several years, and when his experience proved that it was of great value, particularly in the treatment of caries in children's teeth, he at once gave the profession the benefit of his deductions, at a considerable sacrifice, too, of time and money spent in attending distant society meetings for that purpose.

There is every possible incentive for dentists residing in large cities to engage in some form of original work, but when a man's lot is cast in a small town, as Dr. Stebbins's was, and he rises superior to the discouraging and non-appreciative influences surrounding him, and forces world-wide recognition from scientific men, the credit due him is certainly very great.

Dr. Stebbins was prominent in the different dental societies of his vicinity, and for a time president of the old Connecticut Valley Dental Society.

His death makes a vacancy in the ranks of the profession which cannot be filled.

*The President.*—Now for a few moments we will ask attention to the work of the committees. Dr. Bogue, the chairman of the Committee on Operative Dentistry, being absent, we will be glad to hear from any member of the committee.

*Dr. George A. Wilson.*—Being one of the committee on operative dentistry, whose report is now called for, and in the absence of its chairman, let me say, pursuant to the subject of the evening,—that of reducing the pain of our operations,—instead of a cataphoric instrument, which I have not tried, I have been using, for the past five years or more, chloroform applied on a pellet of cotton in the cavity, after placing on the rubber dam. It takes little time, acts rapidly, and very satisfactory results follow.

*The President.*—Will Dr. Wilson tell us how long a time is required to get the result?

*Dr. Wilson.*—From five to fifteen minutes may be required to reduce extreme sensitiveness. It hurts a little at first, but if the chloroform is applied *warm*, the sensitiveness soon subsides. If there are two cavities open at the same time, the application can be made to one while the other is operated upon. In this way absolute *dryness of the cavity* is secured,—the secret, perhaps, of pain reduction,—while the suggestion conveyed to the mind by slight inhalation also has, upon most patients, a seductive and soothing effect. It is a simple thing, and has proved very efficient, more so than any other remedy that I have tried.

*The President.*—We will now have the pleasure of listening to Dr. Wendell C. Phillips. He has no need of an introduction, for he has been with us before.

*Dr. Wendell C. Phillips.*—Mr. President and Gentlemen, I hope you will bear with me if I call your attention to what appears to have been an error in the report which your Committee on Current Literature published in the July number of the INTERNATIONAL DENTAL JOURNAL. It so happened that in that issue an article appears from my own pen, in which I make certain statements which are contradicted by your committee on current literature. I know you will do me the honor to give me an opportunity to defend the position which I take, and at the same time I know that, so far as your society is concerned, you are after the truth in all matters pertaining to cataphoresis. Now, in the article to which I refer I make the following statement: "A rheostat is an adjustable resistance introduced into the circuit in simple connection with the patient to control the quantity of current flowing, without regard to steady voltage. An adapter is an elaborated rheostat for use with the various street currents. A cell-selector is a series of contact points, over which travels a sliding switch-arm, each point connecting with one or more cells of a series battery. These cells ordinarily represent about two volts of pressure each." "The fractional volt-selector will give gradations of less than one-quarter of a volt," etc.

Now, gentlemen, I want to say that by actual test the fractional volt-selector will give not only gradations of less than one-quarter of a volt, but less than one-tenth of a volt. The report of your committee, to which I refer, says as follows: "Drs. Morton and Gillett bring forward the Wheeler volt-selector, an apparatus for turning on the current slowly; it is misnamed a volt-selector, and is merely a suitable rheostat."

Now, either your committee is at fault or Professor Morton, Dr. Gillett, and myself are at fault in that statement. Feeling sure that you would like to know which one is correct, I asked Mr. Wheeler to-night to bring the apparatus necessary to prove by ocular demonstration that the ground taken by Professor Morton, Dr. Gillett, and myself is the correct one. I might further say that it was Professor Morton's suggestion that gave the name of fractional volt-selector to this instrument. I do not know of any one who has had anything to do with electro-therapeutics who is more justly entitled to give a name to such an instrument than Professor Morton. The instrument referred to is not a rheostat, because it controls voltage in fractions.

The subject of the evening, "A Table of Differential Resistances of Fluids for Cataphoric Study," was then taken up, and an interesting discussion ensued, being participated in by Drs. Wendell C. Phillips, George S. Allan, J. Morgan Howe, Harry Waite, Mr. George M. Wheeler, and the President.

As the subject is to be continued at a future meeting a full report will be published at that time.

Adjourned.

S. E. Davenport, D.D., M.D.S.,
*Editor The New York Institute of Stomatology.*

